# Trends and disparities in the surgical management of spinal fractures in Sweden during 2008–2023

**DOI:** 10.1186/s12891-025-08313-8

**Published:** 2025-01-17

**Authors:** Niklas Barle, Michael Axenhus

**Affiliations:** 1 Danderyd Hospital, Department of Orthopaedic Surgery, Stockholm, Sweden; 2 Department of Clinical Sciences at Danderyd Hospital, Karolinska Institutet, Stockholm, Sweden

**Keywords:** Spinal fracture surgery, Disparities, Future trends, Sweden

## Abstract

**Background:**

Spinal fractures are a group of complex injuries whose management varies according to a number of factors. The aim of this study was to analyze trends in the management of spinal fracture surgery in Sweden from 2008 to 2023 with a focus on disparities based on gender, surgery method, age and geographical location. A secondary aim was to predict future incidence of spinal fracture surgeries.

**Methods:**

Comprehensive open source data was obtained from the Swedish National Board of Health and Welfare. The data was stratified based on gender, surgery method, age and geographical location per year and analyzed for trends. Future trends were projected using regression modeling. The student’s T-test was used to compare means.

**Results:**

The incidence of spinal fracture surgery decreased overall from 2008 to 2023 while maintaining an increased incidence for males compared to females. The highest incidence for osteosynthesis was 2.7 per 100 000 inhabitants in 2008 and 1 in 2023. This trend is projected to be reversed in 2030. Several surgery methods have decreased in usage and are projected to reach close to 0 in 2030. The patient group that underwent spinal fracture surgery had a higher mean age in 2023 compared to 2008. The incidence of spinal fracture surgery varied significantly across Sweden where region Östergötland performed 6.3 surgeries per 100 000 inhabitants and region Örebro performed 1.4.

**Conclusions:**

We found several trends where males may undergo spinal fractures surgery more commonly than females. Probable influencing factors may be increased life-spans and osteoporosis. This may also explain the observed shift towards older age groups in spinal fracture surgery. The decreased use of several surgery methods may reflect changes in operational techniques, demographics, and more standardized care. Geographical disparities may indicate different local health care protocols and uneven healthcare utilization and access.

**Trial registration:**

Not applicable.

**Supplementary Information:**

The online version contains supplementary material available at 10.1186/s12891-025-08313-8.

## Background

Spinal fractures include a multitude of injuries from simple fractures of the spinal processes to complex fractures involving multiple vertebrae. The incidence of spinal fractures can be generally described as a bimodal trend with high energy trauma in the young and osteoporosis related trauma in the elderly [1]. Spinal fractures are associated with high morbidity, mortality and disabilities, where the latter may be most prominent [2]. In some cases, surgical fixation is necessary to enable faster recovery and to achieve correct alignment in the spine. Sequelae is common and often has secondary effects such as increased mortality and high healthcare costs. The use of surgery for spinal fractures varies internationally [3].

Surgical management of spinal fractures includes a number of different procedures. The most commonly used methods include external fixation, osteosynthesis and various combinations thereof. External fixation indicates the use of Halo-vests, i.e. fixation of the skull via screws to an orthosis that is fixed to the torso via a vest, for treating unstable cervical spine fractures. The choice of surgical method is based on fracture stability, patient factors and available technology [4]. The management of spinal fractures has changed over the years based on emerging new developments in surgical techniques, better preventative measures and significant demographic shifts [3, 5]. Internationally, these trends may affect people differently based on socioeconomic status and reflect broader changes in how healthcare policies are applied [6]. Mapping the trends of spinal fracture incidence in a large population is useful in order to guide stakeholders and clinicians in developing targeted interventions and proper resource allocation. There is a lack of nationwide studies examining trends in surgical management of spinal fractures. Understanding recent development in this field is necessary to enable potent, cost-effective and equal ways of managing spinal fracture surgeries in the future [7].

The aim of this study was to examine trends in the incidence of spinal fracture surgery over a 15 year period in Sweden with a focus on demographic changes. The secondary aim of the study is to predict future changes in the surgical management of spinal fractures.

## Methods

### Ethical considerations

The data used in this study is publicly available and anonymized. Therefore, ethical approval and informed consent were not needed. Clinical trial number is not applicable.

### Study setting

The Swedish National Health Service guarantees healthcare access for all Swedish citizens, providing subsidized emergency treatment, general hospital care, and outpatient services. Every resident of Sweden is given a unique and permanent Swedish personal identification number, which remains with them throughout their life or until they emigrate. This identification number is essential for all interactions with public or private healthcare systems and is recorded in all national healthcare registers. Sweden is divided into 21 administrative regions. These regions play a critical role in managing healthcare, public transportation, and regional development. Each region is governed by a regional council, elected by the residents, and is responsible for providing a range of public services including healthcare.

### Data collection

This is a retrospective cohort study that utilizes data from the National Patient Register (NPR) of the Swedish National Board of Health and Welfare’s National Patient Register (SNBHW) [7]. The NPR is validated for the use in epidemiological and retrospective studies [8]. The NPR is based on the International Statistical Classification of Diseases and Related Health Problems version 10 (ICD-10) and corresponding Classification of Healthcare Procedures (CHP) codes. Data extracted included age, sex, region and surgical technique. We included the CHP-codes of external fixation (NAJ29), osteosynthesis (NAJ49,NAJ69 and NAJ79) and combined (NAJ89) or unspecified method (NAJ99). We also included the CHP-codes for osteosynthesis which we included into a single group (NAJ49,NAJ69 and NAJ79).

### Study population

#### Inclusion criteria

1. Individuals with residency in Sweden at one point during 1st of January 2008 and 31st of december 2023.
2. Individuals who underwent spinal fracture surgery with external fixation (NAJ29), osteosynthesis (NAJ49, NAJ69 and NAJ79), combined method (NAJ89) and unspecified method (NAJ99).

#### Exclusion criteria

1. Spinal fracture surgery due to pathological fracture, i.e. fractures associated with malignancy.
2. Fracture revision surgery.
3. Osteotomies or ligament repair surgeries.

### Data analysis

The overall annual incidences of each procedure were calculated per 100 000 inhabitants to account for potential changes in population [9]. The data was stratified based on surgery method, sex, age group and region to allow for comparisons of trends over time. The incidence rates were calculated by dividing the number of surgical procedures according to total population as reported by Statistics Sweden [9]. Significant differences between populations was determined using the student’s T-test. For predictions regarding future trends regression analysis was used. We fitted exponential, linear, logarithmic, polynomial and potential regression models to each incidence trend line. Predictive analysis was performed on the best fitted model for each incidence trend. 95% confidence intervals (CI) were used where applicable. A *p*-value of < 0.05 was considered significant.

## Results

**Fig. 1:**
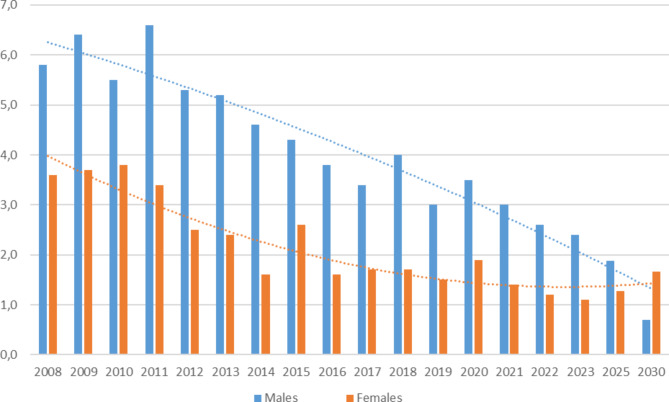
Number of spinal fracture surgeries performed in Sweden. Incidence is per 100 000 inhabitants, year and sex. Male and female incidence are indicated by blue and orange respectively . Trendlines indicate projected incidence for 2025 and 2030

The incidence of spinal fracture surgery in Sweden has decreased since 2008 (Fig. 1). The incidence per 100 000 for males in 2008 was 5.8 and in 2023 2.4 (*p* = 0.28). The incidence per 100 000 for females was 3.6 in 2008 and 1.1 in 2023 (*p* = 0.14).

The incidence of spinal fracture surgery showed a trend, which was not statistically significant, of being more commonly performed in males throughout all observed years. For example, the incidence was 6.6 for males and 3.4 for females in 2011 (*p* = 0.40) and 4.6 for males and 1.6 for females in 2014 (*p* = 0.20). Future trend analysis shows that spinal fracture surgery in males will decrease while surgery incidence in females might increase, eventually surpassing the male incidence (Fig. 1).

While the number of performed surgeries decreased overall, the difference between males and females was consistent between surgical methods when comparing the incidence between 2008 and 2023 (Supplementatary Table 1, Supplementary Fig. 1).

**Fig. 2:**
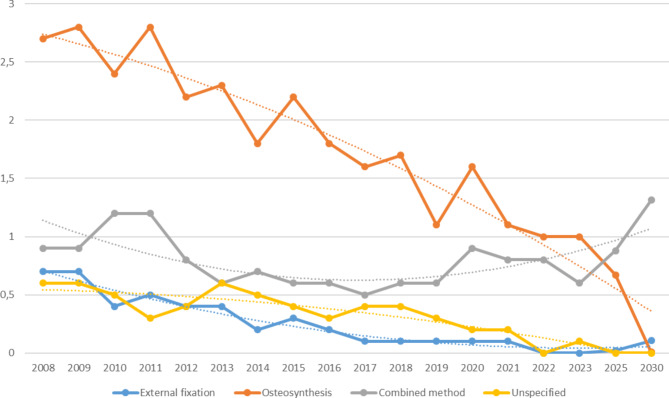
Incidence of spinal fracture surgery methods per 100 000 inhabitants and year. Trendlines indicate projected incidence for 2025 and 2030

The incidence of spinal fracture surgery using osteosynthesis, external fixation and unspecified method in Sweden has decreased over time (Fig. 2). In 2008 the total occurrence of spinal fracture surgeries in Sweden was 529, and in 2023 259 surgeries. The incidence per 100 000 for osteosynthesis was 2.7 in 2008 and 1 in 2023 (*p* < 0.01). For external fixation it was 0.7 in 2008 and 0 in 2023 (*p*-value < 0.01). The incidence for unspecified method was 0.6 in 2008 and 0.1 in 2023 (*p* < 0.01). The incidence for combined method remained relatively unchanged between 2008 and 2023 (*p* = 0.48). The incidence for external fixation, osteosynthesis and unspecified method are projected to decrease and reach close to 0 over the coming years, while the incidence of combined method is projected to increase.

**Fig. 3:**
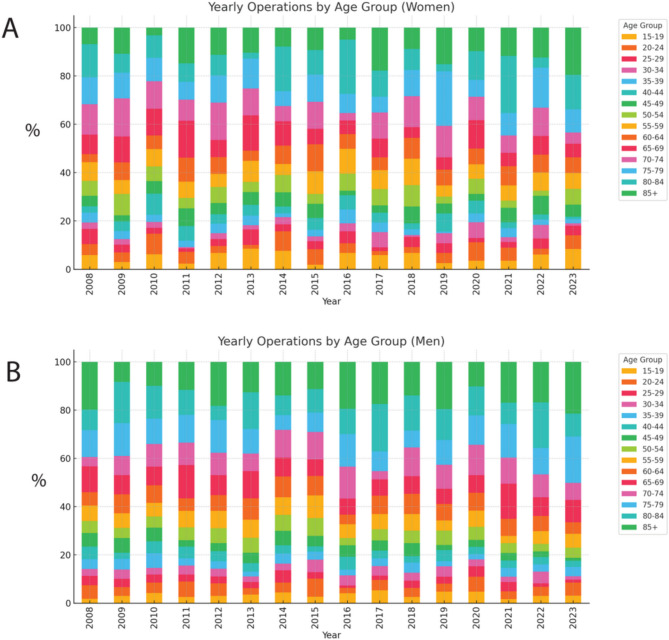
Number of spinal fracture surgeries for females (**A**) and males (**B**) respectively, per 100 000 inhabitants, year and age group. Age groups shown as percent per age group during 2008 to 2023. Age groups are displayed in 5 year intervals

The incidence per age group fluctuated over time with a trend towards more older patients during 2023 compared to 2008, particularly amongst the oldest age groups above 75 years of age in women (Fig. 3A) compared to men(Fig. 3B).

**Fig. 4:**
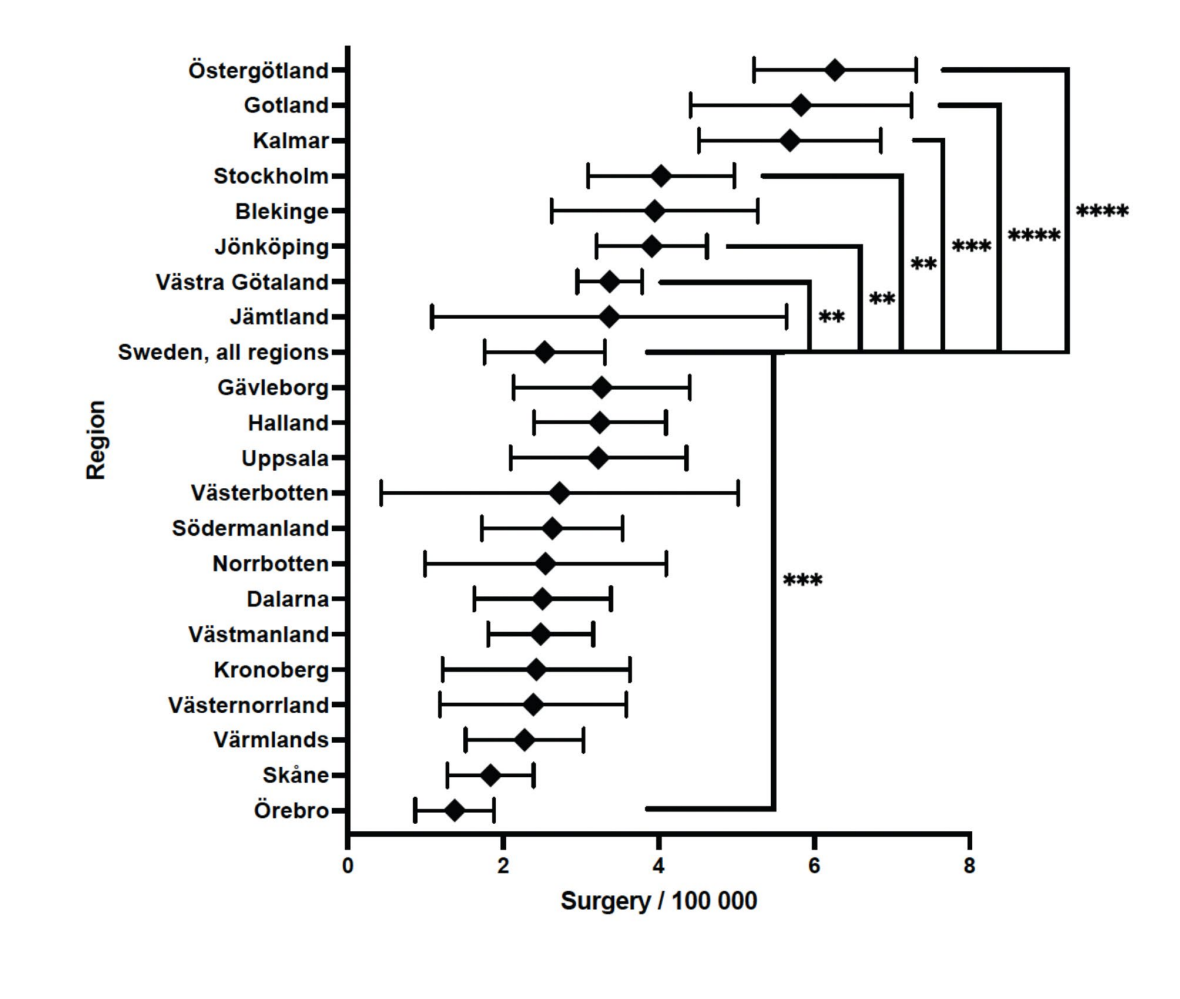
Mean regional incidence of spinal fracture surgeries during 2008 to 2023. Median value is displayed with 95% confidence intervals. Comparative analysis show significant difference between national average and regions with *<0.05, **<0.01, ***<0.001 and ****<0.0001

The incidence of spinal fracture surgery differs markedly across Sweden. For example, region Östergötland performed 6.3 surgeries per 100 000 inhabitants which is well above the national average of 3,3, while the corresponding number for Örebro region was 1.4, well below the national average (Fig. 4).

## Discussion

The incidence of spinal fracture surgery decreased steadily across the study period. Furthermore, males underwent spinal surgery more frequently than females across all observed years, although the differences did not reach statistical significance. These trends align with earlier studies and may be explained by the fact that males are more commonly involved in trauma [10].

Interestingly, our future trend projections indicate that the incidence for spinal surgery in males will decrease while the incidence for females will increase and even surpass the incidence for men in 2030. One of the reasons for this may be a shift in population demographics, as the average life-span will increase over the coming years, especially in females. This in turn may lead to an increased incidence of osteoporosis and therefore also of osteoporosis-related spinal fractures [5]. Another possible reason is socioeconomic developments where females may be more commonly exposed to trauma and occupational hazards traditionally associated with males [11].

There was a significant decrease in the usage of external fixation, osteosynthesis and the use of unspecified surgical methods in Sweden between 2008 and 2023 whereas the use of combined methods remained relatively unchanged. The overall incidence of spinal fracture surgery may have decreased due to a number of factors. Firstly, advancements in non-operative fracture treatment such as bracing, pain medication and physiotherapy have made conservative treatment a viable option in many cases [12]. Secondly, over recent years the use of advanced imaging techniques such as computer tomography and magnetic resonance imaging has become more common. Thus clinicians are able to assess fracture stability more precisely and therefore reserve operative treatment for cases where it is absolutely necessary [13]. Thirdly, evolution of evidence based clinical guidelines have shifted towards non-operative management of spinal fractures where the risks associated with the surgery are more severe than the potential benefit of the procedure [2].

External fixation has traditionally been used mainly in cases of severe fracture instability and/or in patients with multiple associated injuries. This mainly concerns the use of halo-vests in cervical spine injuries. In our study it is predicted that the use of external fixation will approach 0 in 2025. However, it is unlikely that external fixation completely disappears as a surgical method as it will be usable in certain niched cases. Its use may have decreased as a result of the development of techniques that enable more exact reconstruction of spinal fractures [13–15]. Osteosynthesis in spinal fracture surgery has seen several improvements over the past few years including techniques minimizing soft tissue damage and blood loss, optimization of the use of pedicle screws and utilization of intraoperative computer tomography [2, 16]. Still, the incidence of this procedure declined over the past 15 years and is projected to decrease further in 2025 and 2030, although it is unlikely to reach 0 as our projections imply. The GHP code group of “unspecified method” includes a number of less common techniques. The decrease and predicted future further decrease of unspecified methods may reflect a more standardized healthcare system with less need for experimental procedures [13]. Furthermore, a more extensive use of “second opinions” in healthcare centers specialized in the fracture in question may contribute to this development [17].

The use of combined methods remained unchanged over the observed period and is predicted to increase over the coming years. This may reflect its continued usability in cases demanding a multifaceted approach. Individualized surgical procedures appear to play a role in certain cases in both past, present and future [13, 15].

The incidence of spinal fracture surgery displays a shift towards older age groups in recent years. This is in line with global trends with aging populations and therefore an increase in osteoporosis and osteoporosis-related fractures [5]. Similar trends have been observed in other studies [18, 19]. This implies the need for better understanding of underlying factors, preventative measures and treatment options. Taken together with our findings of decreased overall incidence, our data suggest that spinal fracture surgery patients are getting both older and likely more unsuited for surgical intervention.

The incidence of fracture surgery differed remarkably between Swedish regions. There were severe regional differences where Västra Götaland, Jönköping, Stockholm, Kalmar, Gotland and Östergötland showed significantly higher than average incidence of spinal fracture surgery, while Örebro had a significantly lower incidence. A possible explanation for this is the way healthcare is organized in Sweden with major tertiary centers localized in the largest cities. Hospitals in Västra Götaland, Östergötland and Stockholm serve as referral centers for other regions which may skew the incidence [20]. Another possible explanation is the varying availability of healthcare professionals, including surgeons, medical facilities, rehabilitation units etc. If surgery is more readily available as a treatment option, it may be utilized more commonly [21]. In contrast, the incidence in Örebro was significantly lower than the national mean. Possible explanations for this include differing medical guidelines, different population characteristics or lack of surgical expertise. Regional differences in treatment of spinal disorders have been indicated in earlier studies [22, 23]. These differences suggest the need for a more standardized approach to spinal injuries to make sure that healthcare in Sweden is effective and equal regardless of geographic location.

The findings of this study have implications for clinicians involved in the management of spinal fractures. The observed trends highlight the importance of adapting surgical practices to an aging population which may necessitate a shift towards more conservative treatment options. Additionally, the regional disparities in surgical incidence underscore the need for standardized care protocols across Sweden to ensure equitable treatment outcomes. Clinicians should also be aware of the predicted decrease in the use of certain surgical methods, emphasizing the need for continued education and training in newer, more effective techniques that align with these trends.

There are several limitations in our study including its retrospective design and reliance on registry data which may be lacking in accuracy when it comes to coding. Also, there may be other confounding factors such as varying socioeconomic conditions and availability of healthcare. These limitations could potentially be counteracted by more detailed studies incorporating patient comorbidities and regional socioeconomic factors. Future studies should also focus on long term outcomes and how to implement these findings in the clinical setting. We chose to exclude pathological fractures and fractures that underwent revision surgery because of the special challenges these conditions entail. Malignancy-related fractures have different pathophysiological mechanisms, patient populations and treatment options [24]. Revision surgeries have a variability in previously performed surgeries and risk for confounding factors [25]. By excluding these two groups we aimed to provide a clearer picture of trends in primary operative management of spinal fractures in Sweden.

## Conclusion

This study highlights notable trends and disparities in spinal fracture surgeries in Sweden from 2008 to 2023. Overall, the incidence has decreased, with a shift towards older patients and a persistent higher rate among males, though this may reverse by 2030 due to demographic changes. The decline in certain surgical methods suggests a move towards conservative management, while regional disparities indicate differences in healthcare access and protocols. Addressing these disparities and adapting to an aging population will be crucial for optimizing future spinal fracture care in Sweden.

## Electronic supplementary material

Below is the link to the electronic supplementary material.

Supplementary Material 1: Table 1

Supplementary Material 2: Figure 1

## Data Availability

The datasets generated and analyzed during the current study are available in the Swedish National Board of Health and Welfare’s National Patient Register (SNBHW), (https://www.socialstyrelsen.se/en/statistics-and-data/registers/national-patient-register/).
