# Supplementary material for: Trends and disparities in the surgical management of spinal fractures in Sweden during 2008–2023
Source: BMC Musculoskelet Disord. 2025 Jan 17;26:62. doi: 10.1186/s12891-025-08313-8 (PMC11744951; doi:10.1186/s12891-025-08313-8)
Supplement: Supplementary file 2 — Supplementary Material 2: Figure 1 [file 12891_2025_8313_MOESM2_ESM.pdf]

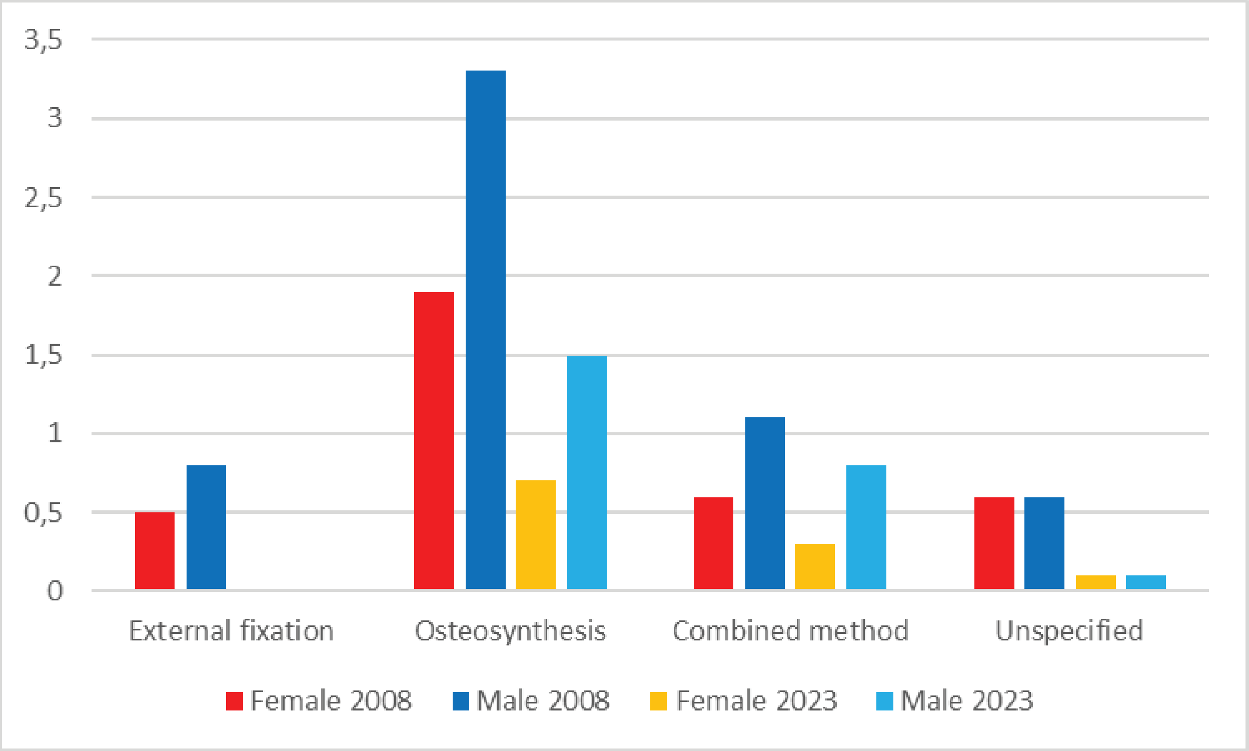

Supplementary Figure 1. Number of spinal fracture surgeries performed per 100 000 inhabitants, sex, and surgery method in 2008 and 2023.
